# Intestinal Histomorphometric Alterations During Experimental Strongyloidiasis in Mice Treated with *Rosmarinus officinalis* Essential Oil

**DOI:** 10.1007/s11686-026-01378-y

**Published:** 2026-08-19

**Authors:** Lorena Q. A. Tanaka, Kamilla A. M. Dutra, Nicolas M. H. da Silva, Izadora M. Assis, Gabriela A. C. Duarte, João B. A. Souza, Eleuza R. Machado, Ana Paula S. Perez, Rosângela M. Rodrigues

**Affiliations:** 1 Biomedicine Course, Institute of Health Sciences, Federal University of Jataí (UFJ), Jatobá Campus, BR 364, Km 194, number 3800, Jataí, GO 75801-615 Brazil; 2 Graduate Program in Applied Health Sciences, Institute of Health Sciences, Federal University of Jataí (UFJ), Jataí, Goiás Brazil; 3 Medicine Course, Institute of Health Sciences and Graduate Program in Biosciences and One Health, Institute of Agricultural Sciences, Federal University of Jataí, Jataí, Goiás Brazil; 4 Anhanguera College of Brasília, Brasília, Federal District Brazil

**Keywords:** *Strongyloides venezuelensis*, Experimental infection, *Rosmarinus officinalis* essential oil, Intestinal mucosa, Goblet cells, Histomorphometry

## Abstract

**Purpose:**

Strongyloidiasis is an intestinal helminthiasis that can induce structural alterations in the intestinal mucosa during infection. The present study evaluated histomorphometric alterations of the small intestine in mice experimentally infected with *Strongyloides venezuelensis* and treated with *Rosmarinus officinalis* essential oil.

**Methods:**

Infected animals were treated orally with *R. officinalis* essential oil (200 or 400 mg/kg), ivermectin or control treatments. Histomorphometric parameters of the small intestine were evaluated, including villus height, epithelial height, crypt depth, muscular layer thickness, brush border height and goblet cell counts. Parasitological parameters were also assessed to characterize the infection model.

**Results:**

Histomorphometric analysis demonstrated that treatment with essential oil at 200 mg/kg was associated with recovery of villus and epithelial height, increased brush border and goblet cell numbers, as well as attenuation of muscular layer hypertrophy, suggesting preservation of mucosal structure during infection. The 400 mg/kg dose, although associated with greater reduction in parasite burden, was accompanied by increased crypt depth, indicating more intense proliferative remodeling of the intestinal mucosa. Ivermectin promoted the greatest reduction in parasite burden with marked elimination of eggs and females.

**Conclusion:**

These findings indicate that *R. officinalis* essential oil modulates intestinal mucosal architecture during experimental strongyloidiasis and highlight its potential influence on host intestinal responses during infection.

**Supplementary Information:**

The online version contains supplementary material available at 10.1007/s11686-026-01378-y.

## Introduction

Human strongyloidiasis, caused by the helminth *Strongyloides stercoralis*, is a chronic neglected tropical disease characterized by autoinfection and affecting approximately 600 million people worldwide, particularly in developing countries in tropical and subtropical regions [1]. The autoinfective cycle enables a potentially lifelong infection, often with absent or transient symptoms [2]. Clinical manifestations may range from mild to moderate gastrointestinal disturbances to severe outcomes, including hyperinfection syndrome and disseminated strongyloidiasis in immunocompromised individuals, conditions associated with high mortality rates [3].

In the host, the parasite lodges mainly in the duodenal crypts, triggering a Th2-type immune response involving eosinophils and inflammatory cytokines [4]. This interaction between parasite and host tissue may induce structural alterations in the intestinal mucosa, including epithelial damage, villus shortening and crypt remodeling, which can compromise mucosal integrity and nutrient absorption. Persistent inflammation and tissue alterations associated with infection, including inflammatory infiltrate in the submucosa and lamina propria, have been described as relevant parameters for understanding disease pathogenesis and host-parasite interactions [3, 5].

Conventional treatment of strongyloidiasis is based on synthetic anthelmintics such as benzimidazoles and ivermectin, the latter being the drug of choice in non-disseminated infections because of its high efficacy and good tolerability [6, 7]. However, the use of ivermectin may be associated with adverse effects, and the widespread and sometimes indiscriminate use of anthelmintics in different settings, including mass drug administration programmes and the marked increase in ivermectin consumption reported in recent years, has raised concerns about the potential emergence of parasite resistance, particularly in light of observations in veterinary medicine [8–10].

Given this context, the search for effective and safe complementary therapeutic approaches has highlighted the potential benefits of phytotherapeutic agents in the management of parasitic diseases [11–13]. *Rosmarinus officinalis* (rosemary) has attracted attention due to its antioxidant, anti-inflammatory and antiparasitic properties demonstrated in experimental studies [14–16]. Its essential oil, rich in oxygenated monoterpenes, has been reported to modulate inflammatory responses and protect gastrointestinal tissues, in addition to exhibiting activity against different helminths and protozoa [17–19].

However, current literature focuses predominantly on in vitro antiparasitic activity and lacks in vivo studies investigating the integrated effects of rosemary essential oil in models of intestinal helminth infections. To investigate the progression of strongyloidiasis in vivo, the experimental model using *Strongyloides venezuelensis*, a rodent parasite, is widely employed due to its biological and immunological similarity to human infection and its ease of maintenance under laboratory conditions, allowing therapeutic strategies to be evaluated in a controlled environment [20].

In this context, the aim of the present study was to evaluate histomorphometric alterations of the intestinal mucosa in mice experimentally infected with *S. venezuelensis* and treated with *Rosmarinus officinalis* L. qt. cineol essential oil. The study integrates parasitological parameters with quantitative analysis of intestinal architecture in order to investigate possible modulatory effects of the essential oil on host intestinal tissue during infection. To date, no studies have evaluated the in vivo effects of *R. officinalis* essential oil on intestinal histomorphometric parameters in a *Strongyloides* infection model, highlighting the gap addressed by the present work.

## Materials and Methods

### Animal Ethics Committee

All experimental procedures involving animals were approved by the Ethics Committee on the Use of Animals of the Federal University of Jataí (UFJ), Brazil (protocol no. 002/2023). All procedures were conducted in accordance with the guidelines of the National Council for the Control of Animal Experimentation (CONCEA) for the care and use of laboratory animals [21].

### Study Site

The experiments were conducted at the Parasitology and Histopathology Laboratories of the Federal University of Jataí (UFJ), Jataí, Goiás, Brazil.

### Essential Oil Source

The essential oil of *Rosmarinus officinalis* L. (cineole chemotype) used in this study was purchased from a commercial supplier (Terra Flor Aromaterapia, Brazil). The oil was obtained by steam distillation of the aerial parts of the plant and supplied in amber glass bottles. The product corresponded to batch no. 23,045 and was used according to the manufacturer’s specifications. According to the supplier’s certificate of analysis, the major constituents included 1,8-cineole (eucalyptol), limonene, α-pinene, and camphor, characterizing the oil as a cineole chemotype (Supplementary File S1).

### Animals

Adult male Swiss albino mice, aged 8–10 weeks and weighing approximately 25–35 g, were used. The animals were obtained from the central animal facility of the Federal University of Goiás (UFG) and maintained at the animal facility of the Federal University of Jataí (UFJ), Jataí, Goiás, Brazil, under controlled conditions and specific pathogen-free status. Throughout the experimental period, all animals received the same standard commercial rodent diet and filtered water ad libitum. The diet was maintained unchanged during infection and treatment. Animals were then assigned to experimental groups according to the study design.

### Obtaining Filariform Larvae

As an experimental model of strongyloidiasis, the rodent parasite *Strongyloides venezuelensis* was used due to its biological and immunological similarities to human infection [20, 22]. The parasite was maintained and amplified in gerbils (*Meriones unguiculatus*) at the animal facility of the Federal University of Jataí (UFJ), which served as the source of infective larvae.

Fecal samples from infected gerbils were cultured on animal mineral charcoal for three days at 28 °C in a BOD type incubator. Filariform (L3) larvae were subsequently recovered using the Rugai et al. (1954) technique: briefly, fecal samples were wrapped in gauze and placed in contact with warm water (40–45 °C), allowing the larvae to actively migrate to the bottom of the vessel, where they were collected and counted under light microscopy [23].

### Experimental Infection and Treatment

For infection, mice were anesthetized with isoflurane (2% in oxygen) and inoculated subcutaneously in the abdominal region with 1,500 filariform (L3) larvae of *S. venezuelensis* per animal.

Treatment began 24 h after infection and continued once daily until day 6 post-infection (6 dpi). Treatment regimens were previously standardized based on in vitro studies that indicated better performance of the concentrations of 200 and 400 mg/kg for rosemary essential oil, which was diluted in phosphate-buffered saline (PBS) containing 1% dimethyl sulfoxide (DMSO). Ivermectin was diluted in water immediately before administration and used as a positive control at a dose of 0.2 mg/kg, administered orally by gavage.

All treatments were administered orally by gavage in a volume adjusted to body weight, once daily at the same time, and animals were not fasted prior to treatment.

Animals were randomly allocated into five experimental groups (*n* = 6 per group): Control (non-infected and untreated mice); GI: Infected (infected and untreated mice); GII: Infected/Ivermectin (infected mice treated with a single dose of ivermectin); GIII: Infected/OE 200 (infected mice treated daily with rosemary essential oil [OE] at 200 mg/kg); and GIV: Infected/OE 400 (infected mice treated with rosemary essential oil at 400 mg/kg daily). Treatments for the essential oil groups were administered daily for six days, whereas the ivermectin group received a single dose 24 h after infection.

On day 7 post-infection, fecal samples were collected, followed by deep anesthesia and euthanasia by cervical dislocation for collection of small intestine samples. Cervical dislocation was performed only after confirming an adequate depth of anesthesia, based on the absence of spontaneous movement and response to stimulation. The procedure was carried out by trained personnel, and death was confirmed before tissue collection.

The experiment was performed in two independent runs to assess reproducibility. Both runs showed the same overall pattern of response across the experimental groups, with no differences that altered the biological interpretation of the findings. One complete experimental series (*n* = 6 animals per group) was used for formal statistical analysis, while the second run was used to confirm the consistency of the observations.

### Assessment of Parasite Burden

Parasite burden was determined using three parameters: egg counts per gram of feces, number of parthenogenetic females in the intestine, and parasite fecundity. Egg counts per gram of feces (EPG) were estimated using the McMaster technique, as described by Rodrigues et al. [24]. For egg counts per gram of feces (EPG), animals were placed in individual cages, and fecal samples were processed using a saturated NaCl solution, filtered, and transferred to McMaster chambers for manual counting under light microscopy. The number of parthenogenetic females was determined after euthanasia on day 7 post-infection (7 dpi). The initial portion of the small intestine from all animals in each group (*n* = 6 per group) was removed, longitudinally opened, and incubated in saline solution at 37 °C for two hours in Petri dishes. The females present in the intestinal mucosa were then counted under a stereomicroscope. Parasite fecundity was calculated as the ratio between the number of eggs excreted per gram of feces and the total number of females recovered from the intestine of each animal.

### Histomorphometric Analysis

After euthanasia, the small intestine from each animal was dissected and divided into duodenum, proximal jejunum, distal jejunum and ileum. Each segment was longitudinally opened and fixed in 10% buffered formalin. The samples were processed by dehydration in graded alcohol, clearing in xylene and embedding in paraffin to obtain histological blocks. Sections of 5 μm thickness were cut using a manual microtome and mounted on slides, followed by staining with hematoxylin and eosin (H&E) and periodic acid–Schiff (PAS), according to the protocols of the Histopathology Laboratory of the Federal University of Jataí (UFJ).

For morphometric analysis, H&E-stained sections were used to measure villus height (100× magnification), crypt depth, muscular layer thickness, villus epithelial height (400× magnification) and brush border height (1000× magnification). For villus height and crypt depth, 30 measurements were obtained per intestinal segment for each animal. The remaining parameters were evaluated using a total of 200 measurements per group. Measurements were performed using Image-Pro Plus 6.0 software.

Histological and morphometric analyses were performed by a single observer blinded to the allocation of the experimental groups. Goblet cells were counted in PAS-stained sections in 30 representative fields per tissue section at 400× magnification [25].

### Statistical Analysis

Data were expressed as mean ± standard deviation (SD). Statistical analyses were performed using GraphPad Prism software (version 8.1). Data normality was assessed using the Kolmogorov–Smirnov test. Variables that did not follow a normal distribution were analyzed using the Kruskal-Wallis test followed by Dunn’s multiple comparison test. Data with normal distribution were analyzed by one-way analysis of variance (ANOVA). Differences were considered statistically significant when *p* < 0.05.

## Results

### Quantitative Histomorphometric Parameters of the Small Intestine

Quantitative histomorphometric parameters of the small intestine are summarized in Table 1. Infection with *S. venezuelensis* did not significantly reduce villus height in any intestinal segment compared with the non-infected control group, although treatment-related differences were observed in specific regions of the small intestine. In the duodenum, animals treated with *R. officinalis* essential oil at 200 mg/kg showed greater villus height than ivermectin-treated animals (*p* < 0.0001), whereas in the proximal jejunum, OE 400 mg/kg was associated with increased villus height compared with the control (*p* < 0.001), infected untreated (*p* < 0.001), and infected/ivermectin groups (*p* < 0.0001).

Infection was associated with reduced villus epithelial height in all intestinal segments relative to the non-infected control group. Ivermectin treatment was associated with additional reductions in most segments, whereas OE 200 mg/kg was associated with higher epithelial cell height in the small intestine. Muscular layer thickness also varied among groups. Infection increased this parameter in the duodenum (*p* < 0.0001), while ivermectin was associated with greater muscular thickness in some intestinal segments; this pattern was attenuated in the essential oil-treated groups.

Crypt depth showed comparatively limited variation among groups. Nevertheless, OE 400 mg/kg increased crypt depth in the distal jejunum (*p* < 0.0001) and in the duodenum and ileum (*p* < 0.001) compared with the control group. Brush border height also varied according to intestinal segment. Ivermectin treatment reduced microvillus height in the duodenum (*p* < 0.0001), proximal jejunum (*p* < 0.0001), and ileum (*p* < 0.05), whereas rosemary essential oil treatment was associated with higher brush border height values in several segments.

Goblet cell counts likewise differed among treatments, with lower values in ivermectin-treated animals in several intestinal segments and higher counts in several portions of the small intestine in animals treated with OE 200 mg/kg.

**Table 1:** Intestinal histomorphometric parameters of mice from the experimental groups

Histomorphometric parameters	Experimental groups				
	Control	GI	GII	GIII	GIV
Villus height (µm)					
Duodenal	192.90 ± 36.30^a,b^	195.00 ± 25.27^a,b^	176.10 ± 29.00^a^	223.80 ± 39.91^b^	198.80 ± 42.77^a,b^
Proximal jejunal	166.10 ± 49.56^a^	167.80 ± 38.25^a^	155.30 ± 29.60^a^	180.40 ± 36.28^a,b^	216.10 ± 42.46^b^
Distal jejunal	140.80 ± 48.59^a^	125.10 ± 30.62^a^	138.40 ± 33.50^a^	140.80 ± 32.56^a^	148.00 ± 45.61^a^
Ileal	118.30 ± 21.24^a,b^	127.40 ± 20.60^a^	138.40 ± 33.50^a^	109.90 ± 17.34^b^	119.70 ± 16.00^a,b^
Villus epithelial height (µm)					
Duodenal	12.57 ± 2.55^a^	10.86 ± 2.27^b^	9.83 ± 1.93^c^	13.56 ± 2.40^d^	11.77 ± 1.78^e^
Proximal jejunal	11.65 ± 2.92^a^	10.15 ± 2.29^b^	8.81 ± 2.25^c^	11.63 ± 2.27^a^	10.60 ± 1.93^b^
Distal jejunal	12.14 ± 2.60^a^	8.18 ± 1.90^b^	8.21 ± 1.72^b^	9.52 ± 2.18^c^	9.23 ± 2.22^c^
Ileal	9.64 ± 2.12^a^	8.58 ± 1.72^b^	7.67 ± 1.70^c^	9.47 ± 2.10^a^	7.89 ± 1.60^c^
Muscular layer thickness (µm)					
Duodenal	18.68 ± 7.24^a^	22.97 ± 6.06^b^	22.91 ± 8.39^b^	19.60 ± 5.80^a^	21.08 ± 5.95^b^
Proximal jejunal	17.29 ± 4.71^a^	17.50 ± 5.31^a^	21.46 ± 6.84^b,c^	19.34 ± 4.06^c,d^	20.39 ± 8.57^d^
Distal jejunal	20.43 ± 5.91^a^	20.61 ± 5.90^a^	21.90 ± 8.73^a^	19.51 ± 4.42^a^	22.38 ± 9.10^a^
Ileal	20.00 ± 7.44^a^	18.41 ± 4.45^a,b^	23.00 ± 7.98^a,b^	18.70 ± 4.03^a,b^	21.29 ± 7.10^b^
Crypt depth (µm)					
Duodenal	28.40 ± 7.30^a^	35.00 ± 10.23^b^	33.77 ± 5.26^c^	30.83 ± 6.38^d^	38.79 ± 14.92^a^
Proximal jejunal	31.68 ± 7.15^a^	34.79 ± 7.03^a^	33.71 ± 7.27^a^	38.24 ± 10.48^a^	39.27 ± 12.85^a^
Distal jejunal	29.03 ± 8.06^a^	31.65 ± 5.85^a,b^	31.89 ± 5.98^a,b^	31.25 ± 6.13^a^	35.73 ± 6.46^b^
Ileal	28.82 ± 6.19^a^	30.62 ± 5.90^a,b^	30.30 ± 4.72^a,b^	30.62 ± 9.03^a,b^	35.08 ± 6.88^b^
Brush border height (µm)					
Duodenal	1.15 ± 0.28^a^	1.12 ± 0.40^a^	0.90 ± 0.23^b^	1.74 ± 0.39^c^	1.65 ± 0.62^d^
Proximal jejunal	1.01 ± 0.19^a^	1.32 ± 0.37^b^	1.11 ± 0.27^c^	1.52 ± 0.33^d^	1.65 ± 0.42^d^
Distal jejunal	0.98 ± 0.25^a^	1.08 ± 0.25^b^	1.05 ± 0.23^a, b^	1.49 ± 0.28^c^	1.46 ± 0.41^c^
Ileal	0.96 ± 0.24^a^	1.05 ± 0.21^b^	0.96 ± 0.20^a^	1.45 ± 0.30^c^	1.50 ± 0.32^c^
Goblet cell number					
Duodenal	27.60 ± 8.85^a,c^	25.33 ± 6.85^a^	18.90 ± 7.69^b^	33.53 ± 8.84^c^	25.63 ± 7.15^a^
Proximal jejunal	28.60 ± 7.49^a,c^	23.89 ± 6.17^a,b^	19.57 ± 8.26^b^	34.13 ± 14.05^c^	25.43 ± 8.33^a,c^
Distal jejunal	26.97 ± 7.85^a^	26.03 ± 8.30^a^	19.90 ± 6.00^b^	28.07 ± 7.71^a^	29.30 ± 9.37^a^
Ileal	24.70 ± 7.94^a,b^	28.30 ± 7.95^a^	20.83 ± 7.15^b^	36.47 ± 9.59^c^	27.50 ± 10.04^a,b^

Data are expressed as mean ± standard deviation (SD). Different superscript letters (a, b, c, d) indicate statistically significant differences among groups, whereas values sharing at least one letter are not significantly different (*p* < 0.05)

### Histological Features of the Intestinal Mucosa

**Fig. 1:**
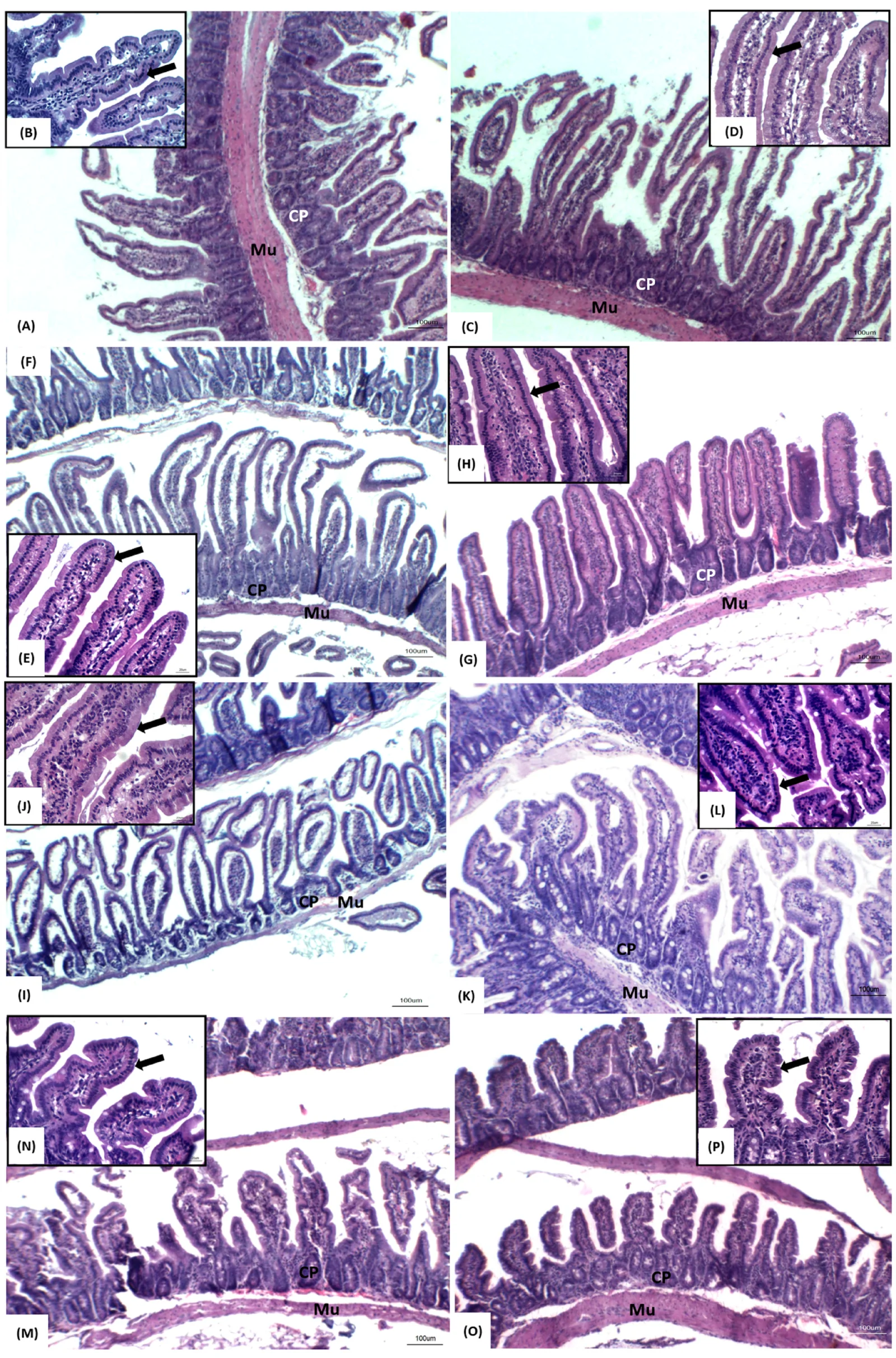
Representative histological sections of the small intestine from mice in the experimental groups stained with hematoxylin and eosin (H&E), illustrating the intestinal structures used for histomorphometric analysis. **A**, **B** GI: duodenal villi. **C**, **D** GI: proximal jejunal villi. **E**, **F** GII: duodenal villi. **G**, **H** GIII: duodenal villi. **I**–**J** Control: proximal jejunal villi. **K**–**L** GIV: proximal jejunal villi. **M**–**N** GII: ileal villi. **O**–**P** GIII: ileal villi. Villus epithelial height (black arrows), muscular layer (Mu), and crypt depth (CP) are indicated in the sections

Representative histological sections of the small intestine stained with hematoxylin and eosin are shown in Fig. 1A–P, illustrating the intestinal structures used for the histomorphometric measurements. Duodenal villi from GI are shown in Fig. 1A, B, those from GII in Fig. 1E, F, and those from GIII in Fig. 1G, H. Proximal jejunal villi from GI, the Control group, and GIV are shown in Fig. 1C, D, I and J, and Fig. 1K, L, respectively. Ileal villi from GII and GIII are shown in Fig. 1M, N, O and P, respectively. Villus epithelial height (black arrows), muscular layer (Mu), and crypt depth (CP) are indicated in the sections. Overall villus architecture was preserved. However, infected animals showed reduced epithelial height compared with the non-infected control group, whereas animals treated with *R. officinalis* essential oil showed epithelial morphology similar to that observed in control animals. Increased muscular layer thickness was more evident in infected animals and in those treated with ivermectin, whereas animals treated with *R. officinalis* essential oil showed a morphological pattern more similar to that observed in the control groups. Greater crypt depth was particularly observed in animals treated with the higher concentration of the essential oil.

### Goblet Cells in the Intestinal Epithelium

**Fig. 2:**
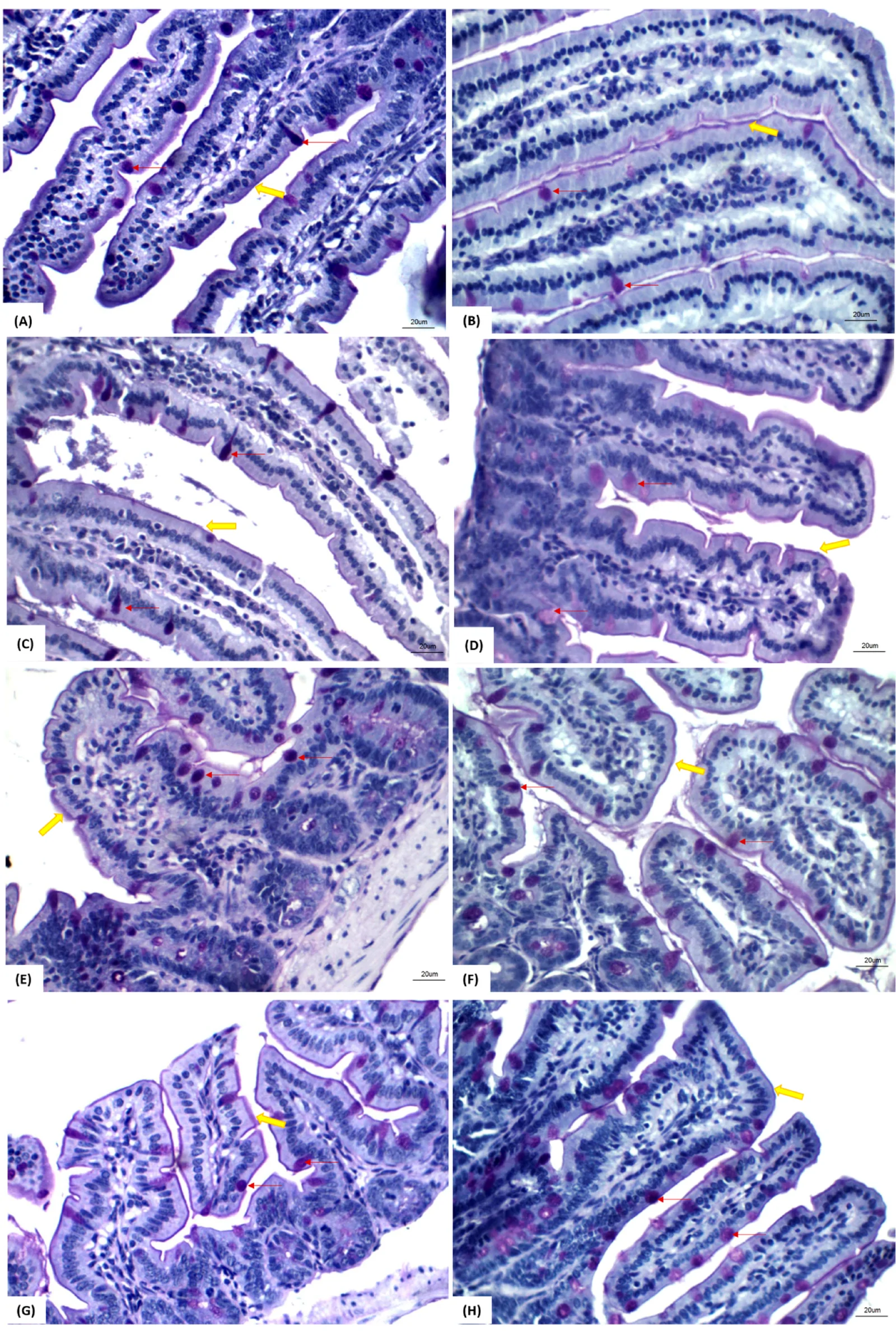
Representative histological sections of the small intestine from mice in the experimental groups stained with periodic acid–Schiff (PAS). **A** GII: duodenal region. **B** GIII: duodenal villus. **C**, **D** GI: proximal and distal jejunal villi, respectively. **E** Control: distal jejunal villus. **F** GIV: distal jejunal villus. **G** GII: ileal villus. **H** GIII: ileal villus. Goblet cells (red arrows) are visible along the villus epithelium, and the brush border (yellow arrows) is located at the apical region of the intestinal epithelium

Representative histological sections illustrating goblet cells in the intestinal epithelium are shown in Fig. 2A–H. The duodenal region from GII and GIII is shown in Fig. 2A and B, respectively; the proximal and distal jejunal villi from GI are shown in Fig. 2C and D, respectively; distal jejunal villi from the Control and GIV groups are shown in Fig. 2E and F, respectively; and ileal villi from GII and GIII are shown in Fig. 2G and H, respectively. Goblet cells (red arrows) are visible along the villus epithelium, and the brush border (yellow arrows) is identified at the apical region of the epithelial lining. In infected animals and in those treated with ivermectin, a reduced number of goblet cells was observed in several intestinal segments compared with the non-infected control group. In contrast, animals treated with *R. officinalis* essential oil at 200 mg/kg showed a greater number of goblet cells along the small intestine. These cells were frequently observed along the villus epithelium in animals treated with the essential oil.

### Parasitological Parameters of Infection

Parasitological parameters were evaluated to characterize the infection model and to complement the histomorphometric analyses (Table 2). Treatment with *R. officinalis* essential oil at 400 mg/kg significantly reduced egg counts compared with the infected untreated group, whereas the 200 mg/kg dose did not differ significantly from the infected untreated group. Ivermectin treatment resulted in the lowest egg counts and number of parthenogenetic females recovered from the small intestine. Parasite fecundity was significantly lower in the ivermectin treated group than in the infected untreated and OE 400 mg/kg groups, while no significant differences were observed between the infected untreated group and the essential oil treated groups.

**Table 2:** Parasitological parameters of mice experimentally infected with *Strongyloides venezuelensis* and treated with *Rosmarinus officinalis* essential oil

Parasitological parameter	GI Infected untreated	GII Ivermectin	GIII EO 200 mg/kg	GIV EO 400 mg/kg
Eggs per gram of feces (EPG)	140,917 ± 26,489^a^	0 ± 0^b^	92,117 ± 13,602^ab^	39,083 ± 13,854^b^
Parthenogenetic females (n)	733.3 ± 172.3^a^	8.7 ± 11.4^b^	714.5 ± 190.2^a^	202.5 ± 51.6^ab^
Parasite fecundity	197.3 ± 36.7^a^	0 ± 0^b^	141.2 ± 57.2^ab^	190.2 ± 34.9^a^

Data are expressed as mean ± standard deviation (SD). Different superscript letters within the same row indicate statistically significant differences according to the Kruskal–Wallis test followed by Dunn’s multiple-comparisons test (*p* < 0.05). Groups sharing at least one superscript letter are not significantly different. GI, infected untreated animals; GII, infected animals treated with ivermectin; GIII, infected animals treated with *Rosmarinus officinalis* essential oil (200 mg/kg); GIV, infected animals treated with *R. officinalis* essential oil (400 mg/kg). Parasite fecundity was calculated as the ratio between eggs per gram of feces and the number of parthenogenetic females recovered from each animal

## Discussion

Infection with *S. venezuelensis*, a well-established experimental model for human strongyloidiasis, reproduced intestinal structural alterations commonly described during infection, including reduced epithelial height and hypertrophy of the muscular layer in specific segments of the small intestine. These changes reflect the interaction between the parasite and the intestinal mucosa as well as host adaptive responses that may involve modifications of the absorptive surface and alterations in intestinal motility [1, 4, 26]. In the present study, treatment with *R. officinalis* essential oil modulated these alterations, with distinct histomorphometric responses according to the administered dose, suggesting an influence of the treatment on intestinal mucosal architecture during infection.

At a dose of 200 mg/kg, the essential oil was associated with a histomorphometric profile indicative of preservation of intestinal mucosal structure. Increased villus height was observed in proximal segments together with recovery of epithelial height, preservation of brush border structure and increased numbers of goblet cells throughout the small intestine. Preservation of villous architecture and epithelial height is particularly relevant in intestinal helminth infections, since these parameters are directly related to nutrient absorption and maintenance of the epithelial barrier. In addition, adequate brush border structure and goblet cell distribution contribute to the protection of the mucosal surface against mechanical and inflammatory damage during infection [27, 28]. The reduction of muscular layer hypertrophy observed in the duodenum and ileum may also indicate attenuation of local tissue responses associated with infection, although inflammatory markers were not evaluated in the present study.

Treatment with the higher dose of the essential oil (400 mg/kg) produced a more complex histomorphometric pattern. Although some favorable effects on villus and epithelial parameters were observed, this dose was also associated with increased crypt depth in several intestinal segments. Crypt hyperplasia is commonly interpreted as an adaptive response related to accelerated epithelial turnover and tissue renewal following villous injury [27]. However, the magnitude of this response requires cautious interpretation, since it may represent either physiological epithelial regeneration or a more pronounced proliferative stimulus associated with tissue remodeling.

The differences observed between doses suggest that the biological responses induced by the essential oil vary according to the concentration administered. While the higher dose showed a greater reduction in parasite burden, the lower dose (200 mg/kg) was associated with a more balanced profile between modulation of infection and preservation of mucosal architecture. In this context, the increased number of goblet cells observed with the 200 mg/kg dose is particularly relevant. Goblet cells play a key role in the intestinal defense barrier by producing mucus that limits direct contact of pathogens and toxins with the epithelial surface and contributes to the maintenance of mucosal homeostasis during intestinal infections [27].

The potential protective effects observed in the intestinal mucosa may be partially explained by biological properties previously described for constituents of *R. officinalis* essential oil, including anti-inflammatory and antioxidant activities attributed to compounds such as 1,8-cineole, α-pinene and camphor [16, 19, 29]. Although these mechanisms were not directly evaluated in the present study, they may contribute to attenuation of structural damage in experimental models of intestinal injury described in the literature [17, 30]. Further investigations including molecular and biochemical markers will be necessary to clarify the mechanisms underlying these observations.

From a parasitological perspective, treatment with the essential oil was associated with reduction in parasite burden, particularly at the dose of 400 mg/kg, which reduced both egg counts and the number of parthenogenetic females recovered from the intestine. However, no significant differences were observed in the fecundity rate of the remaining females, suggesting that the treatment primarily affected parasite burden rather than the reproductive capacity of surviving parasites. Ivermectin remained the most effective drug, producing a marked reduction in egg counts and parasite numbers, consistent with its established role as the drug of choice for strongyloidiasis [6, 7].

Histomorphometric differences were also observed in ivermectin-treated animals, including reduced epithelial height, reduction of brush border height and lower numbers of goblet cells in some intestinal segments, together with increased muscular layer thickness. These findings suggest that structural alterations of the intestinal mucosa may occur under the conditions of the present experimental model. Such observations should be interpreted with caution, particularly in the context of acute experimental infection, but are consistent with reports describing tissue alterations in experimental models exposed to ivermectin, including effects on gastrointestinal, hepatic and renal tissues [31, 32].

Some limitations of the present study should be considered. Histomorphometric analyses were performed at a single time point (7 dpi), which does not allow evaluation of the temporal dynamics of mucosal injury and repair during infection. In addition, the study included only male mice of a single strain and a specific experimental infection model, which may limit extrapolation to other hosts or clinical conditions. The absence of biochemical markers of inflammation or oxidative stress and of functional indicators of intestinal absorption also limits mechanistic interpretation of the observed alterations. Furthermore, although the experimental protocol was replicated, only one experimental series was included in the formal statistical analysis, which may have reduced the potential increase in statistical power associated with larger sample sizes.

Although the present findings demonstrate beneficial effects of *R. officinalis* essential oil on intestinal histomorphometric alterations during experimental strongyloidiasis, translation of these findings to human infection requires additional investigation. Future studies should focus on standardization of the essential oil composition, evaluation of long-term safety, pharmacokinetic characterization and confirmation of efficacy in additional preclinical models of *Strongyloides stercoralis* infection. Furthermore, investigating the potential use of the essential oil as an adjunct to conventional therapy, rather than as a replacement for ivermectin, may represent a promising direction for future research.

In summary, the present findings demonstrate that *R. officinalis* essential oil modulates intestinal histomorphometric alterations during experimental strongyloidiasis. The 200 mg/kg dose was associated with preservation of intestinal architecture and increased goblet cell numbers, whereas the higher dose showed a greater impact on parasite burden but was accompanied by more pronounced remodeling of the proliferative compartment of the mucosa. These observations illustrate how intestinal structural responses may vary according to both parasite presence and treatment conditions, highlighting the relevance of investigating host tissue modulation as part of the host–parasite interaction during helminth infections.

## Conclusion

Treatment with *R. officinalis* essential oil modulated intestinal histomorphometric alterations during experimental strongyloidiasis caused by *S. venezuelensis*. The 200 mg/kg dose was associated with preservation of intestinal architecture, including recovery of epithelial and villus height, maintenance of brush border structure, increased numbers of goblet cells and attenuation of muscular layer hypertrophy in specific intestinal segments. In contrast, the higher dose (400 mg/kg) showed a greater impact on parasite burden but was accompanied by more pronounced remodeling of the proliferative compartment of the intestinal mucosa, as indicated by increased crypt depth.

Ivermectin remained the most effective treatment for parasite elimination in this experimental model, although histomorphometric differences were observed in treated animals. Overall, these findings indicate that *R. officinalis* essential oil influences intestinal structural responses during infection and highlight the relevance of investigating modulation of host intestinal architecture as part of host–parasite interactions during helminth infections. Further studies are needed to clarify the mechanisms underlying these effects.

## Supplementary Information

Below is the link to the electronic supplementary material.

Supplementary Material 1

Supplementary Material 2

## Data Availability

All data generated or analyzed during this study are included in this published article. The chromatographic characterization of the essential oil provided by the manufacturer is available as Supplementary File S1.
