# Supplementary material for: Intestinal Histomorphometric Alterations During Experimental Strongyloidiasis in Mice Treated with Rosmarinus officinalis Essential Oil
Source: Acta Parasitol. 2026 Aug 19;71(5):190. doi: 10.1007/s11686-026-01378-y (PMC13489997; doi:10.1007/s11686-026-01378-y)
Supplement: Supplementary file 1 [file 11686_2026_1378_MOESM1_ESM.pdf]

ULTRA INTERNATIONAL UK LIMITED

Sample Information

Sample Name : **ROSEMARY OIL UI 44702**

Chromatogram

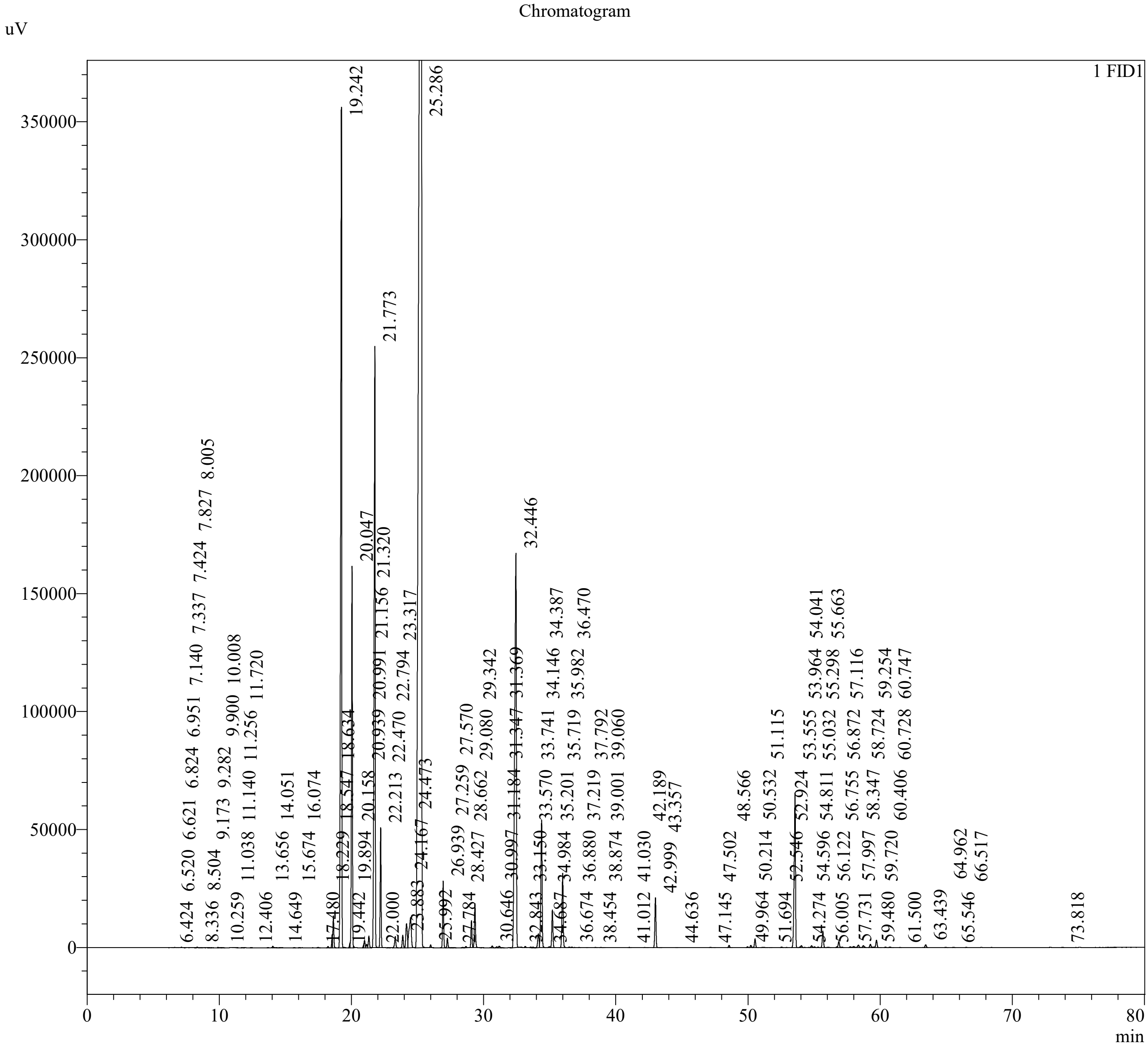

Peak Table

FID1

Ret. Time	Name	Area	Area%
8.336	Isovaleric aldehyde	1091	0.006
8.504	alpha.-Methylbutanal	416	0.002
9.282	2-ethyl-Furan	261	0.001
9.900	2-Methylbutanol	686	0.004
11.038	Toluene	225	0.001
11.256	3-Hexanone	466	0.003
12.406	Octane	350	0.002
13.656	2-Hexenal	358	0.002
14.051	cis-3-Hexenol	3567	0.020
14.649	1-Hexanol	636	0.004
17.480	Bornylene	696	0.004
18.547	Tricyclene	22300	0.127
18.634	alpha-Thujene	67259	0.383
19.242	alpha.-Pinene	2093777	11.937
19.442	Isobutylbenzene	1266	0.007
19.894	alpha-Fenchene	6741	0.038
20.047	Camphene	809053	4.612
20.158	Thuja-2,4(10)-diene	1930	0.011
20.939	3-Octenol	4270	0.024
20.991	3-Octanone	16948	0.097
21.156	Sabinene	8204	0.047
21.773	.beta.-Pinene	1547619	8.823

ULTRA INTERNATIONAL UK LIMITED

Ret. Time	Name	Area	Area%
22.000	2-pentyl- Furan	299	0.002
22.213	Myrcene	245608	1.400
22.470	cis-3-Hexenyl Acetate	275	0.002
22.794	o-Cresyl methyl ether	656	0.004
23.317	.alpha.-Phellandrene	29894	0.170
23.883	delta-3-Carene	31619	0.180
24.167	alpha-Terpinene	79437	0.453
24.473	para-Cymene	148835	0.849
25.286	Eucalyptol + Limonene	9003398	51.329
25.992	trans-beta.-Ocimene	5874	0.033
26.939	.gamma.-Terpinene	146931	0.838
27.259	cis-Sabinene hydrate	21292	0.121
27.570	cis-Linalool oxide	388	0.002
28.427	Fenchone	984	0.006
28.662	para-Cymenene	3340	0.019
29.080	alpha.-Terpinolene	62065	0.354
29.342	Linalool	110942	0.632
30.646	Fenchol	4589	0.026
30.997	.alpha.-Campholenal	2283	0.013
31.184	cis-, para-Menth-2-en-1-ol	4769	0.027
31.347	Nopinone	357	0.002
32.446	Camphor	1511513	8.617
32.843	Isopulegol	1030	0.006
33.150	Camphene hydrate	3285	0.019
33.570	Pinocarvone	1920	0.011
34.146	delta.-Terpineol	42501	0.242
34.387	Borneol	364068	2.076
35.201	Terpinen-4-ol	93540	0.533
35.982	alpha.-Terpineol	198613	1.132
36.470	Myrtenol	1918	0.011
37.219	trans-Piperitol	819	0.005
38.454	Citronellol	882	0.005
38.874	Bornyl formate	759	0.004
42.999	Bornyl acetate	125539	0.716
47.145	Eugenol	1360	0.008
48.566	.alpha.-Cubebene	5849	0.033
49.964	Methyl Eugenol	2499	0.014
50.214	Ylangene	5968	0.034
50.532	alpha-Copaene	23365	0.133
51.115	beta-Bourbonene	2120	0.012
53.555	beta-Caryophyllene	463947	2.645
54.811	trans-.beta.-Farnesene	5327	0.030
55.032	beta.-Copaene	2083	0.012
55.298	.beta.-Copaene	1435	0.008
55.663	trans-.alpha.-Bergamotene	45091	0.257
56.005	Aromadendrene	667	0.004
56.755	.gamma.-Muurolene	1509	0.009
56.872	alpha-Muurolene	17531	0.100
57.731	.gamma.-Muurolene	2217	0.013
58.347	.alpha.-Cadinene	7952	0.045
58.724	.beta.-Bisabolene	6116	0.035
59.254	gamma-Cadinene	9069	0.052
59.480	trans-Calamenene	1712	0.010
59.720	.delta.-Cadinene	21231	0.121
60.406	trans-Cadina-1,4-diene	1296	0.007
		17466682	99.579
