# Supplementary material for: Intestinal Histomorphometric Alterations During Experimental Strongyloidiasis in Mice Treated with Rosmarinus officinalis Essential Oil
Source: Acta Parasitol. 2026 Aug 19;71(5):190. doi: 10.1007/s11686-026-01378-y (PMC13489997; doi:10.1007/s11686-026-01378-y)
Supplement: Supplementary file 2 [file 11686_2026_1378_MOESM2_ESM.docx]

**Supplementary Material**

**Supplementary File S1.** Manufacturer technical report and chromatographic profile of *Rosmarinus officinalis* essential oil (qt. cineole), including batch identification and GC chromatogram provided by the supplier.
